# Exploitation of Thermal Sensitivity and Hyperalgesia in a Mouse Model of Dystonia

**DOI:** 10.3390/life11090985

**Published:** 2021-09-19

**Authors:** Damiana Scuteri, Laura Rombolà, Silvia Natoli, Antonio Pisani, Paola Bonsi, Kengo Hamamura, Giacinto Bagetta, Paolo Tonin, Maria Tiziana Corasaniti

**Affiliations:** 1 Preclinical and Translational Pharmacology, Department of Pharmacy, Health and Nutritional Sciences, University of Calabria, 87036 Rende, Italy; laura.rombola@unical.it; 2 Regional Center for Serious Brain Injuries, S. Anna Institute, 88900 Crotone, Italy; p.tonin@isakr.it; 3 Department of Clinical Science and Translational Medicine, University of Rome Tor Vergata, 00133 Rome, Italy; silvia.natoli@uniroma2.it; 4 Department of Brain and Behavioral Sciences, University of Pavia, 27100 Pavia, Italy; antonio.pisani@unipv.it (A.P.); p.bonsi@hsantalucia.it (P.B.); 5 IRCCS Mondino Foundation, 27100 Pavia, Italy; 6 Laboratory of Chemical Pharmacology, Faculty of Pharmaceutical Sciences, Daiichi University of Pharmacy, Fukuoka 815-8511, Japan; k-hamamura@daiichi-cps.ac.jp; 7 Department of Health Sciences, University “Magna Graecia” of Catanzaro, 88100 Catanzaro, Italy; mtcorasa@unicz.it

**Keywords:** DYT1, torsin A, transgenic mice, neuropathic pain, SNL, heat sensitivity, thermal hyperalgesia

## Abstract

Neuropathic pain is characterized by mechanical allodynia and thermal hyperalgesia to heat, and it affects some 20% of European population. Patients suffering from several neurologic diseases experience neuropathic pain, often finding no relief in therapy. Transgenic mice expressing the gene encoding the human mutant (hMT) or the human wild-type (hWT) torsin A represent a preclinical model of DYT1 dystonia which is the most common form of early-onset inherited dystonia. Baseline thermal sensitivity and hyperalgesia to heat have never been studied in models of dystonia. Therefore, the aim of this research has been to characterize thermal sensitivity in baseline conditions and hyperalgesia to heat after the induction of neuropathic pain through the spinal nerve ligation (SNL) model in mice overexpressing human wild-type and mutated torsin A in comparison to non-transgenic C57BL/6 mice. According to our results, the paw withdrawal latency time to heat in the Hargreaves’ test is significantly lower in the hMT mice (Kruskal–Wallis test = 6.933; *p* = 0.0312*; hMT vs. hWT *p* = 0.0317*). On the other hand, no significant differences in SNL-induced thermal hyperalgesia was found among the three strains (Friedman test = 4.933; *p* = 0.1019). Future studies are needed to better understand the role of torsin A in sensory processing of heat stimuli.

## 1. Introduction

### 1.1. Neuropathic Pain in Neurological Disease Models

Neuropathic pain is a common condition accompanying several diseases, often age-related [1,2], as neuropathies or central pain syndromes [3]. It is frequently undertreated in the course of neurodegenerative diseases [4,5,6] and it is characterized by hyperreactivity to sensory stimulation, resulting in hyperalgesia to mechanical (with also allodynia) and to thermal (heat and cold) stimuli occurring in some 15–50% up to 70% cases [7]. Incidentally, the neurologic *sequelae* of the Coronavirus disease 2019 (COVID-19), caused by the novel severe acute respiratory syndrome coronavirus 2 (SARS-CoV-2), include peripheral neuropathies [8]. Preclinical rodent models are fundamental for the study of human clinical conditions and for the development of therapies [9]. One of the most noteworthy, and still intensely debated, complications with animal models is that they do not always appropriately recapitulate the human disease, thus not translating into clinical settings. Therefore, an accurate characterization of these preclinical models is necessary for their reliability in the study of diseases relevant for human research. Thermal sensitivity to heat and hyperalgesia has been studied in several animal models of neurological human diseases with remarkable global burden.

### 1.2. Thermal Hyperalgesia in Neurological Disease Models

The nociceptive response to heat noxious stimuli has been studied in the double-mutant transgenic TASTPM mice, that represent a preclinical model of familial Alzheimer’s disease (AD) [10]. These mice have shown an increased latency to heat at the age of 6 months, at which they present cognitive deficit [10]. Moreover, thermal hyperalgesia consequent to complete and incomplete Freund’s adjuvant injection (CFA and IFA) has been assessed in a preclinical model of learning and memory impairment induced by bilateral intra-hippocampal administration of beta-amyloid (Aβ)1-40 and Aβ1-42 and, later, of cycloheximide, a protein synthesis inhibitor that prevents memory formation and consolidation [11]. These animals have presented an attenuated development of nociceptive behavior including thermal hyperalgesia, the presence of unaltered baseline thresholds, and an earlier recovery [11]. Thermal sensitivity has also been characterized in parkinsonian mice. In fact, thermal pain hypersensitivity has been described in mice subjected to the unilateral injection of 6-hydroxydopamine (6-OHDA) into the medial forebrain bundle with partial parkinsonism [12]. Moreover, rats administered with 6-OHDA have displayed reduced nociceptive threshold to heat [13]. On the other hand, mice subjected to the administration of MPTP (1-methyl-4-phenyl-1, 2, 3, 6-tetrahydropyridine) to obtain a Parkinson’s disease model display prolonged response latency in the constant temperature, but not in the increasing temperature, hot-plate test that is reduced in the long-term, supporting the development of hyperalgesia [14]. In fact, reduced baseline thermal sensitivity to heat has been found in MPTP PD mice [15]. Thermal hyperalgesia has also been described in MeCP2-308 [16], a mouse model of Rett syndrome i.e., a rare neurodevelopmental disorder caused in the 90–95% of cases by mutations in the methyl-CpG-binding protein 2 (MeCP2) gene [17]. The DYT1 dystonia is a severe early-onset inherited neurologic hyperkinetic movement disorder caused by a GAG deletion in the gene TOR1A of the torsin A [18]. The latter belongs to the “ATPases associated with a variety of cellular activities” (AAA+ ATPase) family [19]. Like the previously analyzed neurological diseases, dystonia is accompanied by pain which occurs in 56–62% of cases [20]. In particular, pain related to adult-onset primary dystonia occurs in the affected region in up to 70% of patients with cervical dystonia (*torticollis*), 30% with blepharospasm and 32% with focal hand dystonia and writer’s cramp [21]. For cervical dystonia it represents the most important factor contributing to worsening of health-related quality of life, causing physical activity limitations in each type of dystonic disturbance [21,22]. Nevertheless, data concerned with mechanical thresholds in clinic are controversial. In fact, pain–pressure thresholds have been reported to be two times lower in patients suffering from idiopathic cervical dystonia and showing a diurnal variation in pain intensity increasing during the day up to a plateau in the evening [23]. On the other side, quantitative sensory testing in patients affected by idiopathic hand dystonia has highlighted an impairment of the sensory system with reduced heat-induced painful stimulation (also demonstrated by contact heat-evoked potentials), as well as a general loss of sensory function including mechanical and pressure pain thresholds [24]. Pain in dystonic patients is not only musculoskeletal, but an alteration of the descending modulation is involved [25]. Moreover, cases of axonal sensory polyneuropathies concurrent with dystonia have been described [26,27]. Botulinum toxin (BoNT) finds application to confer clinical improvement in dystonia due to reduced connectivity with [28] and also to relieve pain [29], as in migraine. However, in the latter, novel approaches with fast action like eptinezumab have been developed [30], although pain in dystonia is not always relieved by BoNT, suggesting that it is not only musculoskeletal in origin and likely implicating the descending inhibitory system [25].

### 1.3. Sensitivity and Neuropathic Pain in Models of Dystonia

Preclinical models of DYT1 dystonia consist of transgenic mice expressing the gene encoding the human mutant (hMT) or the human wild-type (hWT) torsin A [31], presenting slower learning and reduced motor skills at 9 months of age, increased levels of µ opioid receptors, and an accumulation of glutamatergic α-amino-3-hydroxy-5-methyl-4-isoxazolepropionic acid (AMPA) receptors in striatal spiny neurons. All these pathological changes can be implicated in alterations of sensitivity and hyperalgesia. In fact, tapentadol acting as a µ opioid receptor agonist can attenuate heat hyperalgesia in diabetic neuropathic mice [32,33], and the AMPA receptor antagonist perampanel is able to reduce chronic constriction injury-induced heat hyperalgesia [34]. Baseline thermal sensitivity and neuropathic pain-related heat hyperalgesia has never been studied in a preclinical model of dystonia. Therefore, the present research has the purpose to characterize baseline sensitivity and hyperalgesia to heat in the spinal nerve ligation neuropathic pain model in hMT and hWT transgenic mice.

## 2. Materials and Methods

### 2.1. Mice

Three mice strains have been used to study baseline thermal sensitivity and thermal hyperalgesia due to neuropathic pain: (1) C57BL/6J mice (Charles River, catalog number B6JSIFE10SZ-C57BL/6J SPF/VAF;RRID:IMSR_JAX:000664), which are the non-transgenic (NT) mice that have been used as control; (2) mice overexpressing the human wild-type (hWT) torsin A; (3) mice expressing the human mutated (hMT) torsin A at a level comparable to the hWT line [31], i.e., the line hMT1. The hMT mouse model that we have studied is one of several animal models of DYT1 dystonia, which have been generated. These include a homozygous knock-in of the ΔGAG mutation or knockout of torsinA (both of which are lethal at birth); heterozygous knock-in of ΔGAG and transgenic over-expression of mutant human torsinA; and selective knockouts of torsinA in cortex and striatum. Interestingly, none of these mouse models exhibit overt dystonia [31], but many display significant abnormalities of motor behavior or learning, reminiscent of the human non-manifesting carrier state. The transgenic mice (males and females) used in the present study were kindly provided by IRCCS Fondazione Santa Lucia, Laboratory of Neurophysiology and Plasticity, Rome, Italy. Animal breeding and handling were performed in agreement with the guidelines for the use of animals in biomedical research provided by the European Union’s directives and Italian laws (2010/63EU, D.Lgs. 26/2014; 86/609/CEE, D.Lgs. 116/1992). The sample power was calculated by routine formula and setting power to 80% and α = 0.05, according to similar studies in the literature [35] to obtain reliable results while keeping the number of animals as low as possible, based on the 3R approach to refine, reduce, and, at least in part, replace. The experimental procedures were approved by Fondazione Santa Lucia and University Tor Vergata Animal Care and Use Committees, and the Italian Ministry of Health. Mice were housed in groups of four per cage on a 12 h:12 h light dark cycle at a constant room temperature of 22 ± 1 °C and relative humidity of the 65% with food and water *ad libitum*.

### 2.2. Neuropathic Pain Model

The neuropathic pain model is the Spinal Nerve Ligation (SNL) [36]. Animals were anesthetized with 2% isoflurane. According to the model by Kim & Chung, 1992 [36], a midline incision was made in the back skin at L2–S2 level. The left paraspinal muscles were separated from the spinal and transverse processes at L4–S1 level. The left L5 spinal nerve was isolated and tightly ligated with 6–0 silk thread and wound sutured. The sham procedure was identical without the ligation. In agreement with the model, the thresholds of the left injured paw and of the right uninjured paw became different on post-operative day 3. The posture and movements of the hindpaw were monitored during the postoperative days.

### 2.3. Hargreaves’ Test

For the assessment of baseline thermal sensitivity and of paw withdrawal threshold to noxious heat the Hargreaves’ test [37] was used. The behavioral tests were performed twice a day on the 5th and 3rd day before surgery to assess baseline levels of thermal sensitivity normalized to 1. To evaluate thermal hyperalgesia, the tests were carried out on the 3rd, 7th, 14th and 28th postoperative days, since these are the most important time points for the development of thermal hyperalgesia after SNL, that starts from the 3rd day and begins to decrease after 28 days, lasting for 35 days [36]. To perform the Hargreaves’ test, mice were placed in a perspex box for at least 1 h of acclimatation, after which a source of radiant heat (Plantar Test, model 7650, Ugo Basile, Italy) was applied to the surface of the hindpaw with an intensity of 20% and a cut-off of 20 s. The paw withdrawal latency time to thermal stimulation, measured in seconds (sec) was assessed through this test. During the behavioral tests room temperature and humidity were maintained constant.

### 2.4. Statistical Analysis

Data were checked for normality using the Friedman test, being samples < 8, and expressed as mean ± SEM (standard error of measurement) and median + interquartile range (IQR). Statistical differences were assessed through the Kruskal–Wallis and the Friedman test for non-parametric unpaired and paired data, respectively, followed by Dunn’s multiple comparisons test in case of null hypothesis rejection (GraphPad Prism 6). *p* < 0.05 was considered statistically significant.

## 3. Results

### 3.1. Baseline Thermal Sensitivity to Heat of Mice Overexpressing Human Wild-Type and Mutated Torsin A

**Figure 1:**
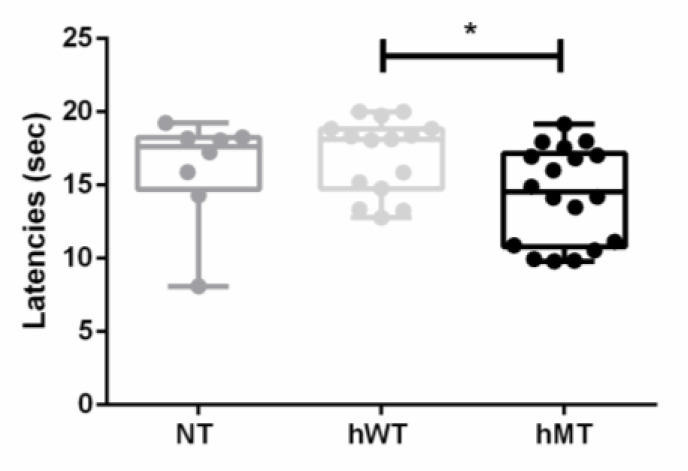
Baseline thermal sensitivity to heat in non-transgenic (NT) mice and in mice overexpressing human wild-type (hWT) and mutated (hMT) torsin A. NT and hWT mice showed comparable, not significantly different, paw withdrawal latency time to thermal stimulation. The hMT mice presented significantly lower paw withdrawal latency time to heat (Kruskal–Wallis test = 6.933; *p* = 0.0312*; hMT vs. hWT *p* = 0.0317*). Data are expressed as median + interquartile range (IQR) of mean latency time in seconds (sec). *p* values < 0.05 are considered statistically significant. n: NT = 8, hWT = 15, hMT = 18. * *p* < 0.05.

The first endpoint of this original research was the characterization of the baseline thresholds of thermal sensitivity to heat in mice overexpressing human wild-type (hWT) or mutated torsin A (hMT), comparing them to the non-transgenic control represented by C57BL/6 mice (NT). The paw withdrawal latency time to heat was significantly lower in the hMT mice (Figure 1; Kruskal–Wallis test = 6.933; *p* = 0.0312*; hMT vs. hWT *p* = 0.0317*).

### 3.2. Thermal Hyperalgesia to Heat of Mice Overexpressing Human Wild-Type and Mutated Torsin A

**Figure 2:**
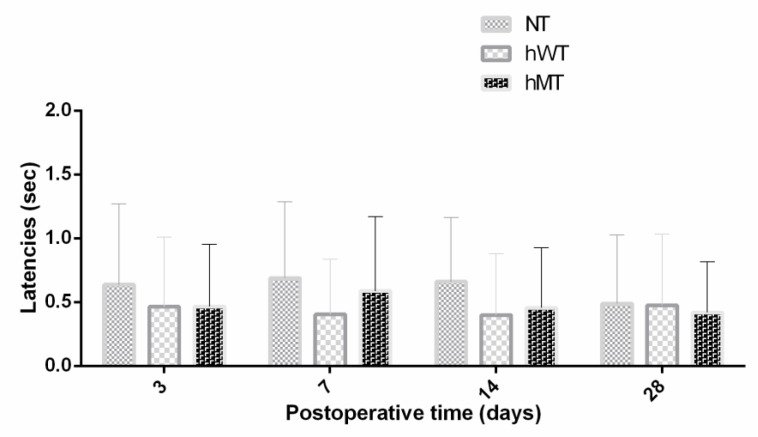
Thermal hyperalgesia to heat in non-transgenic (NT) mice and in mice overexpressing human wild-type (hWT) and mutated (hMT) torsin A. The paw withdrawal latency time to heat did not differ in hWT and hMT mice compared to NT mice (Friedman test = 4.933; *p* = 0.1019). Data are expressed as mean latency time in seconds (sec). *p* values < 0.05 are considered statistically significant. n: NT = 7, hWT = 15, hMT = 18. * *p* < 0.05.

The second endpoint was the characterization of thermal heat hyperalgesia induced by a neuropathic pain model, i.e., the SNL. The overexpression of normal human or mutated torsin A yielded no statistically significant modifications of SNL-induced thermal hyperalgesia to heat compared to NT mice (Figure 2; Friedman test = 4.933; *p* = 0.1019). It is possible to notice that the NT mice presented higher latency times on the 3rd, 7th and 14th postoperative days, in which hyperalgesia peaks, though it is a trend that does not reach statistical significance (Figure 2).

## 4. Discussion

Thermal sensitivity and hyperalgesia to heat has never been characterized in mice overexpressing normal human or mutated torsin A, representing a preclinical rodent model of early-onset inherited DYT1 dystonia. For the first time, the results of this original research work have demonstrated that, although baseline thermal sensitivity is increased in mice overexpressing human mutated torsin A, the mutation has not exerted effect on SNL-induced hyperalgesia. However, a trend toward an increased hyperalgesia is present in hWT and hMT mice compared to NT mice, but it does not reach statistical significance. Previous results from our research group have demonstrated that there are no significant differences in baseline mechanical sensitivity among the three strains [35]. Moreover, the transgenic mice have shown prolonged mechanical allodynia after SNL, suggestive of delayed recovery [35]. Nevertheless, the effect of the human gene on pain processing cannot be excluded [35]. This difference between thermal sensitivity and hyperalgesia and mechanical sensitivity can be due to the diverse sensory information processing and development of mechanical allodynia and thermal hyperalgesia to heat. In fact, thermal hyperalgesia to heat has been suggested to be primary and not secondary, which is attributed to alterations of central pain processing [38], since central sensitization is specific for mechanosensitive pathways [39]. In particular, heat hyperalgesia has been suggested to be absent in the secondary zone, in respect to the injury primary zone, due to lack of facilitation of inputs from heat sensitive nociceptors [39]. In spite of this, thermal hyperalgesia, both in inflammatory and in neuropathic pain, can be a result of peripheral sensitization of fibers expressing transient receptor potential vanilloid 1 (TRPV1) channels and of central mechanisms [7], as demonstrated by the capability of the capsaicin analogue resiniferatoxin to block heat hypersensitivity [40] and by the evidence that it occurs in 10% of patients with central pain [41]. These channels can be involved both in peripheral and in central sensitization, as shown also in aged C57BL/6 mice in formalin test [42]. Torsin A colocalizes with α2δ-1 L-type voltage-dependent calcium channel subunit upregulated during neuropathic pain, in the γ-aminobutyric acid (GABA)-ergic and glutamatergic neurons of the dorsal horn superficial laminae [35] that regulate pain processing to central areas. In particular, torsin A has been associated with disinhibition of striatal GABAergic synaptic activity [43] and GABA-ergic inhibition after nerve injury is necessary to avoid the development of aberrant processing of sensory information within the dorsal horn [44]. Furthermore, mutations of torsin A have been associated with increased level of huntingtin aggregation [45], and it has been observed in Lewy bodies and Lewy neurites in *substantia nigra* and *cortex*, thus likely being implicated in neurodegenerative diseases [46]. In fact, Lewy bodies are a histopathological feature common to several neurodegenerative diseases [47]. However, no immunoreactivity for torsin in Alzheimer’ s disease hippocampus has been reported and it has been detected in association with α-synuclein in Lewy bodies [46]. Incidentally, an association between dystonia and psychiatric and behavioral symptoms, prodromal and occurring in course of neurodegenerative diseases, has been found [48]. Cholinergic dysfunction with significant increase in the vesicular acetylcholine transporter, used to assess cholinergic function in Alzheimer’s disease and Parkinson, has been described in the dorsal striatum of the Tor1a^+/−^mouse model of DYT1 dystonia. Moreover, the possible altered baseline and nociceptive sensitivity threshold can occur in populations with non-specific pain, e.g., chronic nonspecific low back pain [49], likely due to mechanisms of central sensitization. In particular, the thermal threshold is differentially associated with nerve fibre pathology than mechanical as vibration perception threshold [50]. Also, low-threshold thermal nociception to innocuous hot temperatures is not modulated by adaptation as for cool temperatures, supporting the existence of a more complex modulation [51]. Furthermore, chronic constriction injury-induced thermal hyperalgesia but not mechanical allodynia is attenuated by neurotrophin-3 [52]. Thus, altered thermal threshold to heat should be considered in clinic since it might be associated with different responses to the pharmacological treatment of neuropathic pain. These findings need to be applied to the various types of dystonia that can present different sensitive alterations [53,54,55,56]. In fact, in agreement with our results, patients affected by cervical isolated idiopathic dystonia display reduced hot detection threshold [57] at quantitative sensory testing [58].

Therefore, the possible role of the human, wild-type and mutated, gene encoding torsin A in thermal sensitivity and hyperalgesia to heat in the course of neuropathic pain needs to be deeply investigated in future studies, along with pharmacological modulation with α2δ-1 ligands currently used in clinic for the treatment of neuropathic pain.

## 5. Conclusions

This original research has characterized baseline thermal sensitivity and hyperalgesia to heat in the course of neuropathic pain in mice overexpressing human wild-type and mutated torsin A. In spite of an increased baseline thermal sensitivity to heat in hMT mice, no significant differences in hyperalgesia have been found. Further studies are needed to better elucidate the role of human, wild-type and mutated, gene encoding torsin A on thermal stimuli processing.

## Data Availability

The data presented in this study are available within the article.
